# Synergistic photobiomodulation with 808-nm and 1064-nm lasers to reduce the β-amyloid neurotoxicity in the *in vitro* Alzheimer's disease models

**DOI:** 10.3389/fnimg.2022.903531

**Published:** 2022-07-22

**Authors:** Renlong Zhang, Ting Zhou, Soham Samanta, Ziyi Luo, Shaowei Li, Hao Xu, Junle Qu

**Affiliations:** Key Laboratory of Optoelectronic Devices and Systems of Ministry of Education and Guangdong Province, College of Physics and Optoelectronic Engineering, Shenzhen University, Shenzhen, China

**Keywords:** fibrosis amyloid-β (fAβ), microglia, neuroblastoma, photobiomodulation, microglia phenotype, Alzheimer's disease

## Abstract

**Background:**

In Alzheimer's disease (AD), the deposition of β-amyloid (Aβ) plaques is closely associated with the neuronal apoptosis and activation of microglia, which may result in the functional impairment of neurons through pro-inflammation and over-pruning of the neurons. Photobiomodulation (PBM) is a non-invasive therapeutic approach without any conspicuous side effect, which has shown promising attributes in the treatment of chronic brain diseases such as AD by reducing the Aβ burden. However, neither the optimal parameters for PBM treatment nor its exact role in modulating the microglial functions/activities has been conclusively established yet.

**Methods:**

An inflammatory stimulation model of Alzheimer's disease (AD) was set up by activating microglia and neuroblastoma with fibrosis β-amyloid (fAβ) in a transwell insert system. SH-SY5Y neuroblastoma cells and BV2 microglial cells were irradiated with the 808- and 1,064-nm lasers, respectively (a power density of 50 mW/cm^2^ and a dose of 10 J/cm^2^) to study the PBM activity. The amount of labeled fAβ phagocytosed by microglia was considered to assess the microglial phagocytosis. A PBM-induced neuroprotective study was conducted with the AD model under different laser parameters to realize the optimal condition. Microglial phenotype, microglial secretions of the pro-inflammatory and anti-inflammatory factors, and the intracellular Ca^2+^ levels in microglia were studied in detail to understand the structural and functional changes occurring in the microglial cells of AD model upon PBM treatment.

**Conclusion:**

A synergistic PBM effect (with the 808- and 1,064-nm lasers) effectively inhibited the fAβ-induced neurotoxicity of neuroblastoma by promoting the viability of neuroblastoma and regulating the intracellular Ca^2+^ levels of microglia. Moreover, the downregulation of Ca^2+^ led to microglial polarization with an M2 phenotype, which promotes the fAβ phagocytosis, and resulted in the upregulated expression of anti-inflammatory factors and downregulated expression of inflammatory factors.

## Introduction

In recent years, photobiomodulation (PBM), known as low-level light therapy (LLLT), has become popular to treat various neurodegenerative diseases. Usually, red or near-infrared light with a low power density (1–500 mW/cm^2^) is used in PBM to trigger a series of biological responses by influencing the brain activity (Rojas et al., 2012; Song et al., 2012; Huisa et al., 2013; Xu Z. et al., 2017; Zhang et al., 2021). However, the precise mechanism of PBM is yet to be confirmed. According to the most commonly accepted theory, upon absorbing the red or NIR light photons, cytochrome C oxidase (CCO), the terminal enzyme (unit IV) of the mitochondrial electron transport chain, can alter the redox state of the cell to increase the membrane potential as well as the ATP level, which in turn promotes the cell activity to maintain/restore the normal cell function (Chung et al., 2012; Mitrofanis and Henderson, 2020). Moreover, PBM might be effective in regulating the gene expression and activating the signaling pathways (e.g., NF-KB) through the modulation of reactive oxygen species (ROS) levels to influence various physiological processes such as cell signal transmission, enzyme activation, inhibition of cell apoptosis, and promotion of cell differentiation (Chen et al., 2011). Therefore, in the near future, the non-invasive, non-pharmaceutical PBM therapy could become the truly effective means of treating chronic neurodegenerative disorders such as AD which does not have a promising treatment yet.

Microglia are the endogenous immune response cell of the central nervous system (CNS), which plays a vital role in clearing the cell debris and apoptotic neurons with phagocytic function, involving secretion of cytokines and chemokines to maintain the homeostasis (Hansen et al., 2018). However, microglia may not be always protective toward the CNS, as it can induce two different cell phenotypes with opposing cytokine expressions (Miron et al., 2013; Tsay et al., 2013). Microglia will polarize toward an M1 pro-inflammatory phenotype when stimulated by persistent inflammation, leading to the release of neurotoxic inflammatory cytokines such as IL-6 and TNF-α (Olmos and Llad, 2014; Toyama et al., 2021), whereas microglial activation with an M2 phenotype releases some anti-inflammatory cytokines such as IL-4 and TGF-β to induce anti-inflammatory response and tissue repair (Hu et al., 2015). Therefore, maintaining the normal phagocytic function of microglia and promoting the microglial polarization with the M2 anti-inflammatory phenotype could be imperative for the treatment of neurodegenerative diseases. However, in the AD model, wherein the abnormal deposition of Aβ protein is pertinent, the Aβ-induced elevated intracellular calcium ion (Ca^2+^) level also might activate the pro-inflammatory response in microglia (McLarnon, 2005; Chiozzi et al., 2019).

Essentially, AD is a chronic progressive neurodegenerative disease, which may impose a serious threat to human life by gradually promoting abnormal behavior, memory loss, language impairment, cognitive impairment, and overall loss of individuality. Even though millions of older people are affected worldwide due to the AD-related dementia, no promising cure for AD has been invented till date (Wortmann, 2012). The abnormal deposition of Aβ protein has been characterized as a sign of AD that not only causes inflammation by activating microglia to produce neurotoxic pro-inflammatory cytokines, but also can lead to neuronal injury (Hardy and Higgins, 1992; Moreno-Jimenez et al., 2019; Leng and Edison, 2020). The studies also indicated that the injection of fAβ into CNS of animals could induce certain pro-inflammatory traits (Liu et al., 2012; Wirz et al., 2013; Lu et al., 2017), wherein the binding of Aβ protein with the innate immune receptors such as TLR-2 (Liu et al., 2012), TLR-4 (Stewart et al., 2010; Michaud et al., 2013), TLR6, and CD-14 (Landreth and Reed-Geaghan, 2009) perhaps triggered the pro-inflammatory microglial activation. However, microglia essentially play a vital role in regulating brain functions through phagocytosis, leading to the elimination of apoptotic neurons and fAβ along with the pruning of non-functional synapses (Badimon et al., 2020). The studies also revealed that the microglial polarization with the M2 phenotype could effectively reduce the Aβ burden and result in the diminished Aβ-mediated neurotoxicity through the phagocytosis of the deposited Aβ (Tsay et al., 2013). Compelling evidences suggested that PBM with variable irradiation doses could be instrumental in modulating the phenotype of microglia. For instance, von Leden et al. (2013) demonstrated that under high doses of light, BV2 microglia could be polarized to an M1 phenotype, whereas the polarization of BV2 microglia to the M2 phenotype was apparent at lower doses of light. Therefore, PBM with optimized light parameters can provide the wonderful scope of regulating the microglial polarization with a particular phenotype (M2) to attenuate the Aβ burden.

Several studies were conducted in the direction of unraveling the specific mechanism of PBM, involved in microglial modulations. It has been reported that the pro-inflammatory expression, caused by the toll-like receptor, can be suppressed by PBM through the activation of Src-mediated signaling pathway (Song et al., 2012). Therein, post-PBM-treatment microglia witnessed the downregulated expression of the gasotransmitter nitric oxide (NO). In a transgenic mouse model, PBM treatment using the 1,070-nm laser also revealed promising results in reducing the Aβ burden by effectively regulating the microglia to improve the Aβ clearance (Tao et al., 2021). In clinical studies, evidences also indicated the possible neurocognitive recovery in AD upon the 1,064-nm laser irradiation (Vargas et al., 2017). NIR light irradiation not only showed promising results in reducing the fAβ-mediated neurotoxicity through microglial activation, but also could directly promote the viability of neurons by inducing the inhibition of neuronal apoptosis. In this regard, Huang et al. (2014) demonstrated that upon irritation with the 810-nm laser, the high ROS levels of primary cortical neurons can be substantially reduced, which in turn protected neurons from oxidative stress to manifest the neurocognitive recovery in the case of fAβ-mediated neuroinflammation (Li et al., 2016; Hong et al., 2020). Both the proliferation of neurons and their differentiation are the important processes, upholding normal neuronal function. Wu et al. (2021) found that the proliferation of neurons can be efficiently promoted using the 635-nm laser, whereas the 808-nm laser can be employed excellently to improve the differentiation rate of neurons. Based on these results, we envisaged that employing the dual lasers with the wavelengths of 808 and 1,064 nm could synergistically persuade more significant outcomes toward the inhibition of Aβ-induced neurotoxicity than using a single-wavelength laser when implemented in a co-culture system of microglia and neurons.

In this study, we explored the possibility of dual-laser (808 and 1,064 nm, respectively) synergic PBM on the AD model, containing SH-SY5Y neuroblastoma cells and BV2 microglial cells cultured with fibrosis Aβ (fAβ) in a transwell insert system. The biological events, triggered by the microglia and neurons upon irradiation with dual lasers (1,064- and 808-nm lasers, respectively), were studied extensively to realize the potential of this PBM method in reducing the fAβ burden and neurotoxicity in the AD model (D'Andrea et al., 2004; Bolmont et al., 2008; Cherry et al., 2014). It also revealed that the laser irradiation with the 10 J/cm^2^ (a power density of 50 mW/cm^2^) dose and 1,064 nm wavelength can mostly enable the microglial polarization with the M2 phenotype to facilitate the enhanced phagocytosis of fAβ. Moreover, the microglial activation with the 1,064-nm laser irradiation also led to the increased anti-inflammatory attributes as confirmed by the detailed bioimaging studies. On the contrary, the 808-nm laser-mediated (a power density of 50 mW/cm^2^ and a dose of 10 J/cm^2^) PBM evidently enhanced the neuronal activity to effectively inhibit fAβ-mediated neuronal toxicity. The overall study verified the efficacy of PBM on the recovery of neuron viability upon regulating the microglia with the 1,064-nm laser and neurons with the 808-nm laser synergistically in the AD model.

## Results

### 1,064-nm laser promoted the microglial phagocytosis of fAβ

**Figure 1:**
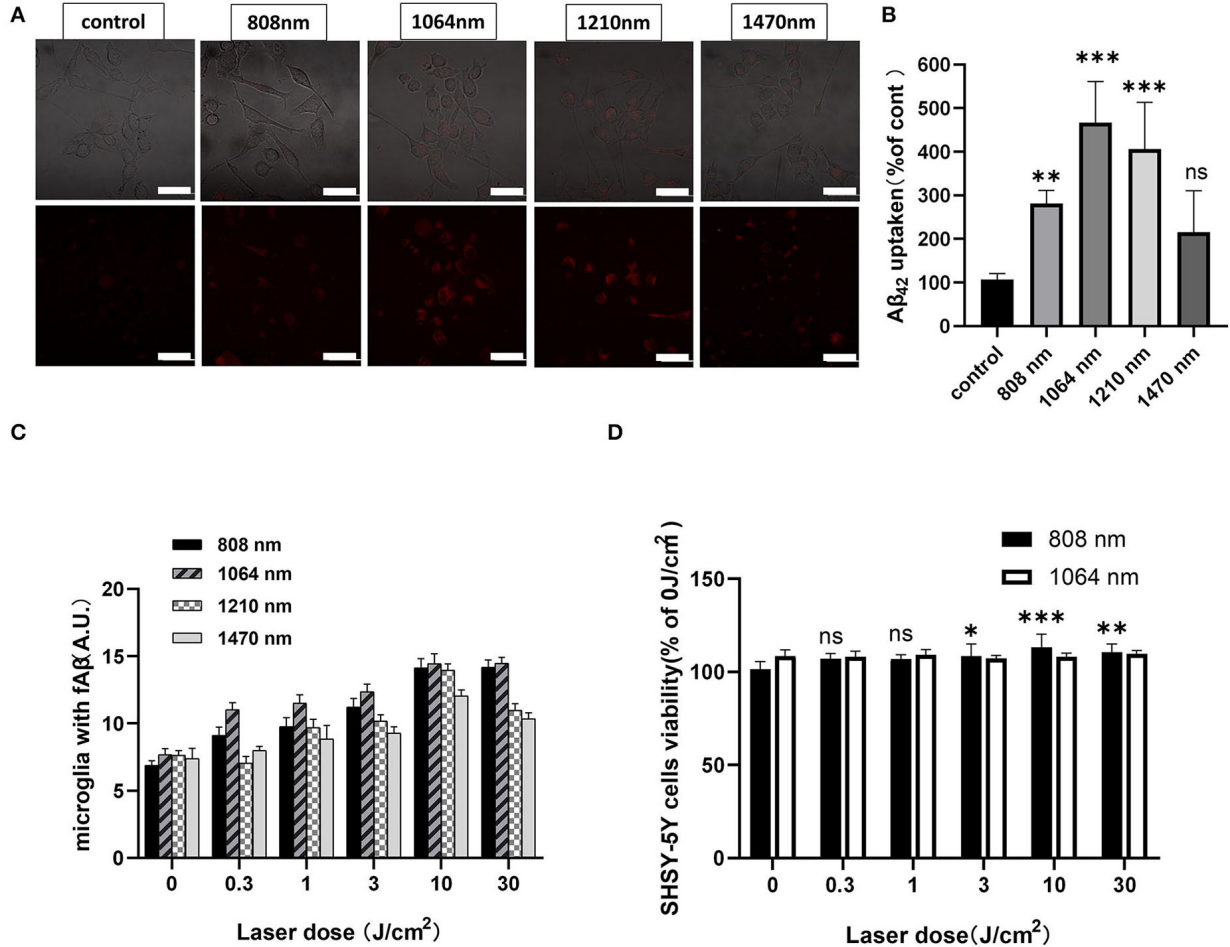
Eight hundred and eight-nanometer laser promoted neuronal activity, and 1,064-nm laser promoted microglial fAβ phagocytosis. **(A)** Confocal fluorescence colocalization imaging for microglia to phagocytosed fAβ after 808-, 1,064-, 1,210-, and 1,470-nm laser PBM treatment; scale bar, 50 μm; **(B)** the quantification of the fluorescence measurements of **(A)**, *n* = 20; **(C)** flow cytometry for fAβ phagocytosis amount by microglia irradiated with the 808-, 1,064-, 1,210-, and 1,470-nm lasers at six different doses, respectively; **(D)** CCK-8 viability assay of SH-SY5Y neuroblastoma cells, *n* = 8. Data in **(B)** and **(D)** are mean ± SEM of at least three independent experiments, *p < 0.05, **p < 0.001, and ***p < 0.0001.

Microglial phagocytosis of fAβ under the 1,064-nm laser irradiation was studied in detail. The representative confocal microscope images are shown in Figure 1A, and the quantification of the fluorescence measurements is shown in Figure 1B. As fAβ can activate microglia to express the pro-inflammatory phenotype and mediate the apoptosis of neurons, it is important to activate the microglia with suitable laser irradiation, so that it could promote the phagocytosis function in the direction of degrading the fAβ directly (Yates et al., 2000; Yao et al., 2005). To figure out which wavelength of near-infrared lasers is the most biologically effective to promote the phagocytosis of fAβ, microglia were irradiated with the 808-, 1,064-, 1,210-, and 1,470-nm lasers. A comparison of the amount of labeled-fAβ uptake upon irradiation with NIR lasers (808, 1,064, 1,210, and 1,470 nm) is presented in Figure 1A. Confocal fluorescence imaging revealed the phagocytic uptake of the most of fAβ by BV2 cells within 4 h of PBM treatment using the 1,064-nm laser. Figure 1B reveals the Aβ uptake for different laser irradiations compared with the control group (808-nm laser group: 158.89%; 1,064-nm laser group: 331.49%; 1,210-nm laser group: 278.42%; 1,470-nm laser group: 101.13%). Moreover, the efficacy of the phagocytosis of fAβ in microglia under different laser doses (0, 0.3,1, 3, 10, and 30 J/cm^2^) was also measured using flow cytometry in quantitative analysis (Figure 1C). It was also observed that the 1,064-nm laser under the 10 J/cm^2^ dose can show the best results in terms of increasing the phagocytosed fAβ content (95.36%) in microglial cells compared with the control group (0 J/cm^2^). Therefore, the overall results validated that the 1,064-nm laser (a dose of 10 J/cm^2^) can induce the most significant response in promoting the phagocytosis of Aβ through the anti-inflammatory microglial activation.

### 808-nm laser restored the viability of neurons

The neuronal cell viability in the CNS is tested to assess whether the brain can show normal functions or not, in terms of cognitive and memory ability. Studying the direct impact of PBM treatment on the viability of neurons is crucial to realizing the therapeutic efficacy. According to the flow cytometry results (Figure 1C), the use of the 808- and 1,064-nm lasers has shown the best responses in promoting the microglia-mediated phagocytosis of fAβ. Similarly, to determine which wavelength in the infrared band is the most effective to protect the neuronal activity, we compared the dose-dependent regulatory effects of the 808- and 1,064-nm lasers on neurons (Figure 1D). SH-SY5Y cells were cultured with fAβ for 24 h before PBM treatment. Subsequently, the neuroblastoma cells were subjected to PBM treatment using the 808- and 1,064-nm lasers. In this experiment, the laser doses of 0, 0.3, 1, 3, 10, and 30 J/cm^2^ were tested at the two wavelengths (808 and 1,064 nm), and the corresponding illumination durations were 0, 6, 20, 60, 200, and 600 s (a power density of 50 mW/cm^2^). The CCK-8 assay-based viability test results revealed that the 808-nm laser had the most significant effect on promoting the viability of SH-SY5Y cells when the 10 J/cm^2^ (*p* < 0.05) laser dose was applied. Therefore, the 808-nm laser could restore the function of neurons to some extent even if the neurons are still experiencing the fAβ-induced toxicity and inflammation.

### 1,064-nm laser regulated the phenotype of microglia

**Figure 2:**
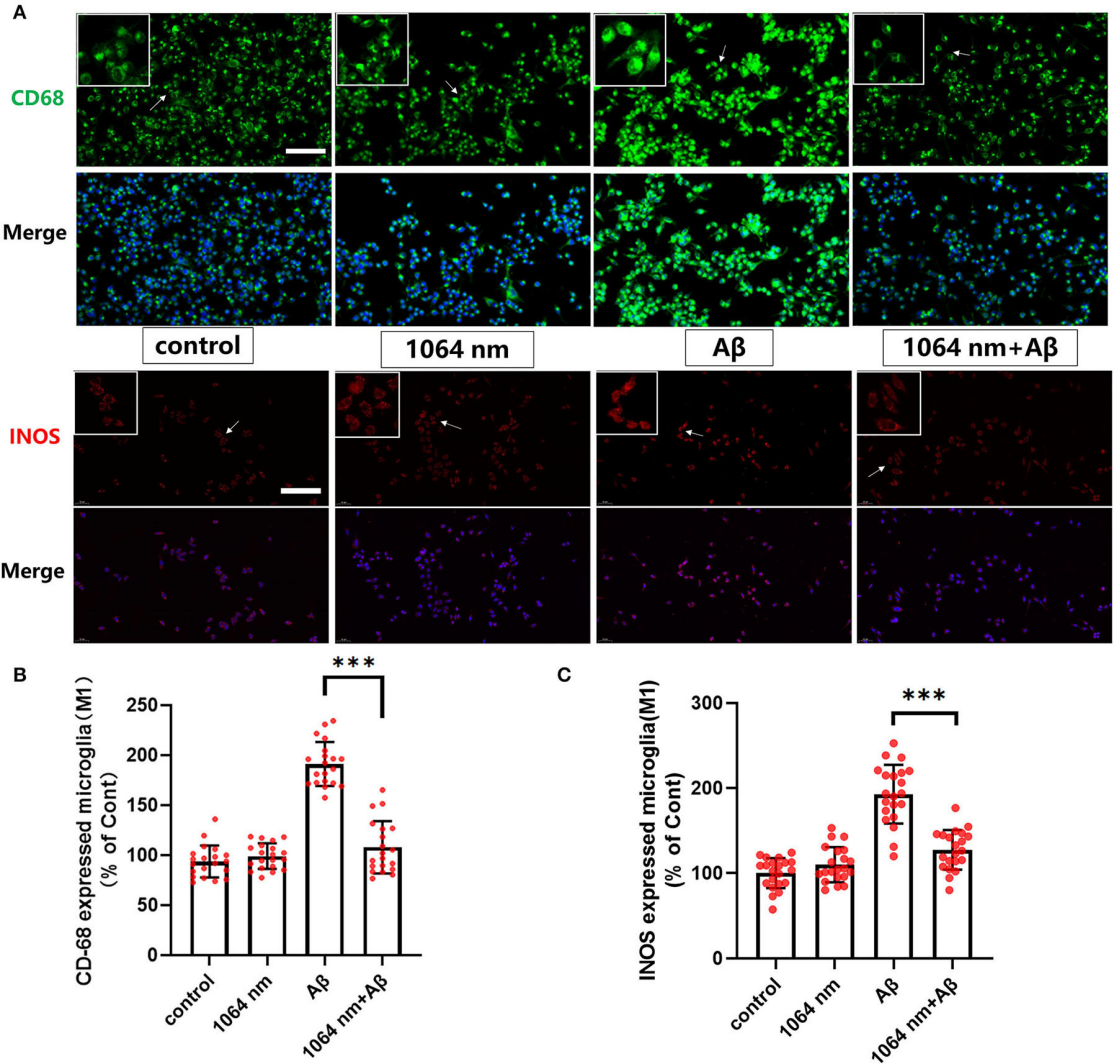
One thousand and sixty-four-nanometer laser regulated the CD68 and iNOS (M1 markers) expression in microglia. **(A)** The fluorescence imaging of CD68 and iNOS after Aβ treatment and 1,064-nm laser irradiation; scale bar, 100 μm; **(B,C)** quantification of the fluorescence measurements. All data are normalized to the control group and shown as the mean ± SEM of at least three independent experiments, ***p < 0.0001.

**Figure 3:**
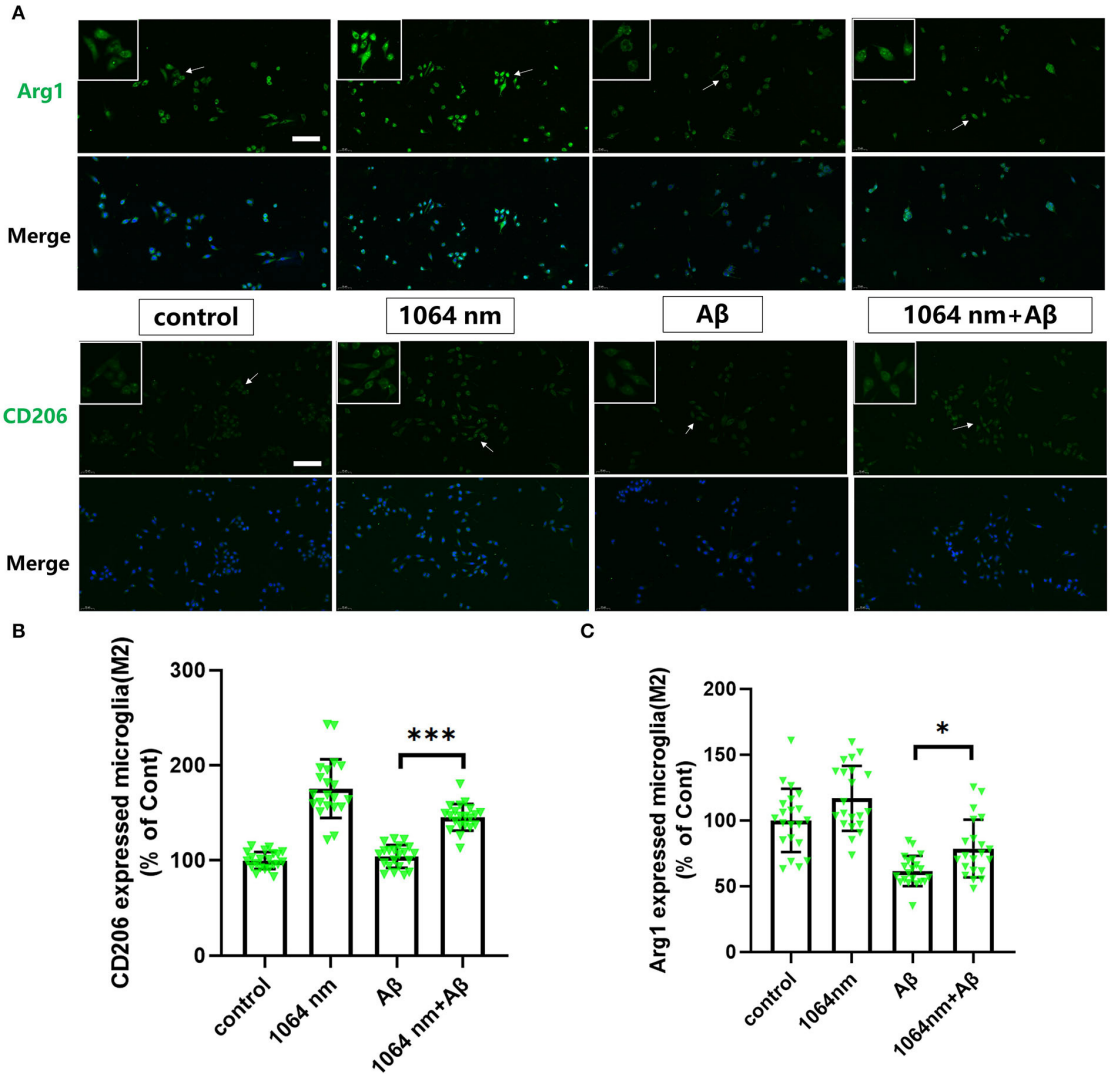
One thousand and sixty-four-nanometer laser regulated the Arg1 and CD206 expression (M2 markers) in microglia. **(A)** The fluorescence imaging of Arg1 and CD206 after Aβ treatment and 1,064-nm laser irradiation; scale bar, 100 μm; **(B,C)** quantification of the fluorescence measurements. All data are normalized to the control group and shown as the mean ± SEM of at least three independent experiments, *p < 0.05 and ***p < 0.0001.

We have already demonstrated that the 1,064-nm laser can promote the microglial phagocytosis, and it is important to explore the reasons behind that. Activated microglia can have two different phenotypes. fAβ can activate microglia to the M1 phenotype which enhances inflammatory expression factors, whereas the microglial activation to the M2 phenotype can facilitate the clearance of fAβ *via* phagocytosis. Therefore, it was prudent to verify whether the 1,064-nm laser can enhance the phagocytosis of fAβ by regulating microglia toward the M2 phenotype or not (Jana et al., 2008). Using immunofluorescence technique, antibodies were fluorescently labeled in microglia, which served as the markers for the M2 (arginase I (Arg1) and CD206) and M1 (inducible nitric oxide synthase (iNOS) and CD68) phenotypes of microglia, respectively. The corresponding confocal microscope images are shown in Figures 2, 3A, and the quantification of the fluorescence measurements is shown in Figures 2, 3B,C. The expression of CD68 (104.01% higher than the control group, Figure 2B and iNOS (92.90% higher than the control group, Figure 2C in microglial cells, cultured with fAβ, was significantly higher than that in the other three groups, which indicated that fAβ has stimulated the transformation of microglia toward the inflammatory phenotype M1. In contrast, when irradiated with the 1,064-nm laser, the expression of CD68 and iNOS in the fAβ-cultured microglial cells returned to the level of control group without a significant difference (*p* > 0.05). These results clearly indicated that the fAβ-induced pro-inflammatory microglial polarization (M1 phenotype) could be inhibited by 1,064-nm (10 J/cm^2^) laser irradiation. In addition, the expression of CD206 and Arg1 in the fAβ-cultured microglial cells was also measured upon the 1,064-nm laser irradiation. The expression of CD206 (75.45%, *p* < 0.0001, Figure 3C and Arg1 (16.79%, *p* < 0.05, Figure 3B) in the 1,064-nm laser-irradiated group was found to be higher than that in the control group, and the fAβ+1,064-nm laser group showed the higher expression of Arg1 (17.03%, *p* < 0.05) and CD206 (41.42%, *p* < 0.001) than that of the fAβ group. Therefore, applying the 1,064-nm laser not only the microglial polarization can be regulated to inhibit the pro-inflammatory phenotype but also it can promote the anti-inflammatory phenotype. The polarization of microglial phenotype can also enhance the phagocytic ability of microglia to reduce the fAβ burden and attenuate the fAβ-induced neurotoxicity of neurons.

### 1,064-nm laser modulated the anti-inflammatory and pro-inflammatory factors in microglia

**Figure 4:**
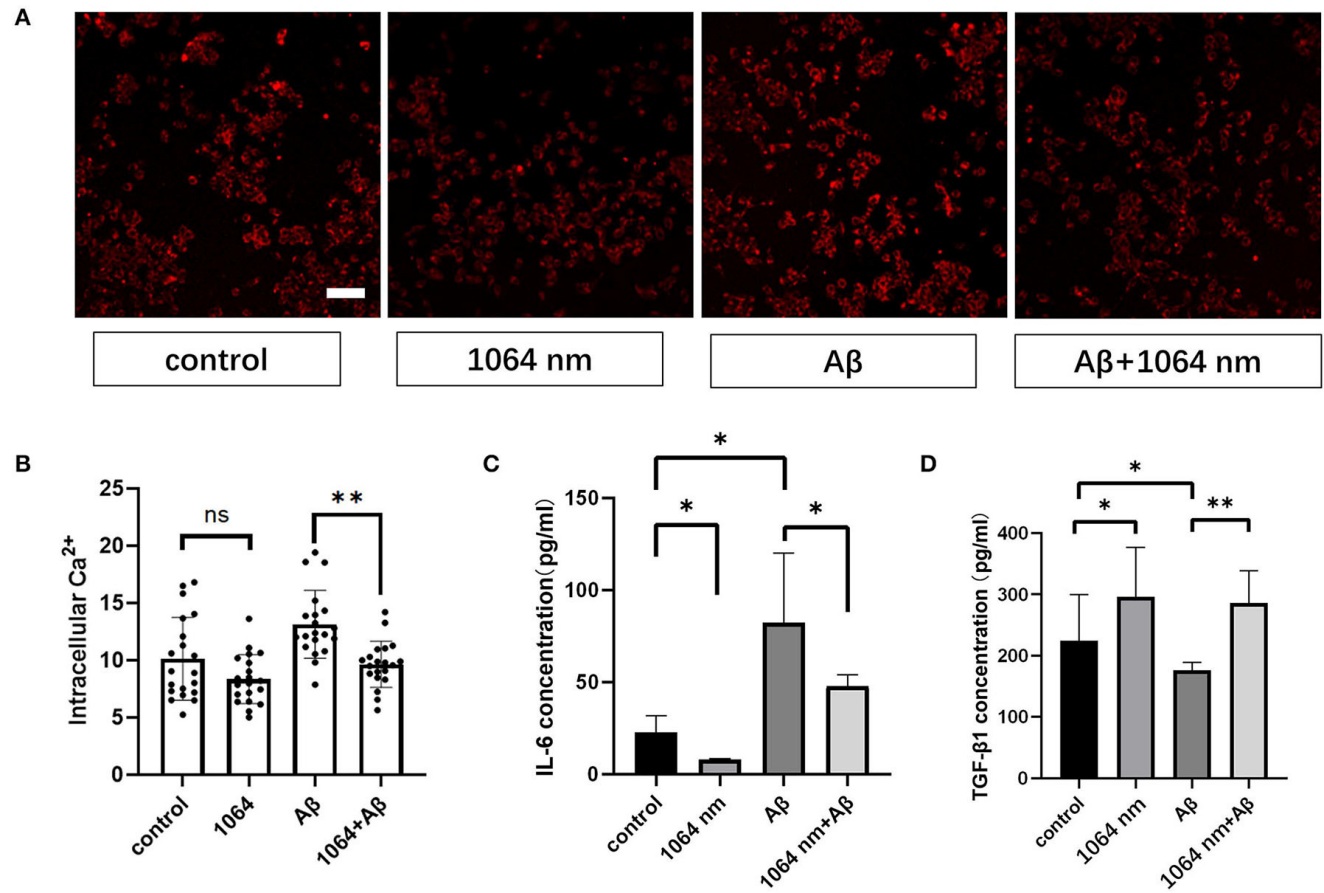
One thousand and sixty-four-nanometer laser regulates cytokines and intracellular Ca^2+^ levels in microglial cells. **(A)** Fluorescence imaging of intracellular Ca^2+^ influx in microglia; scale bar, 100 μm; **(B)** quantification of the fluorescence measurements of images in **(A)**, *n* = 20; **(C,D)** ELISA assay of IL-6 and TGF-β1 of microglia, *n* = 8; data in **(B–D)** are mean ± SEM of at least three independent experiments, *n* = 20, *p < 0.05 and **p < 0.001.

The positive results, obtained from the preceding immunofluorescence study of microglia, encouraged us to investigate whether the inflammatory and anti-inflammatory factors in microglia can be effectively modulated using the 1,064-nm laser or not. IL-6 and TGF-β are the most common inflammatory and anti-inflammatory factors, respectively, in macrophages such as microglia (Paglinawan et al., 2003; Liu et al., 2015). The regulatory effect of the 1,064-nm light irradiation on the inflammatory properties of microglia was evaluated based on the expression of these two kinds of cytokines, which was consistent with the expression of cytokines in M1 and M2 cells as presented in the previous immunofluorescence experiment. Essentially, the experimental groups were divided into four groups similar to the immunofluorescence experiment, wherein the IL-6 and TGF-β expressions were determined by an ELISA kit following the similar fAβ incubation and laser treatment processes. As shown in Figures 4C,D, in comparison with the control group, the IL-6 expression (-64.94%) was downregulated and the TGF-β expression (+63.14%) was upregulated in the 1,064-nm (10 J/cm^2^) group. A similar trend was observed in the 1,064-nm+fAβ group and the fAβ group (Figures 4C,D). Compared with the fAβ group, the contents of IL-6 and TGF-β in the 1,064-nm+Aβ group were decreased by 41.93% and increased by 62.57%, respectively. However, after fAβ stimulation, the expression of IL-6 was significantly upregulated in microglia.

### 1,064-nm laser regulated cellular Ca^2+^ in microglia

Microglial cells almost have no transient Ca^2+^ when they are in the resting state (Eichhoff et al., 2011). Meanwhile, intracellular Ca^2+^ also acts as a secondary messenger in response to inflammation. Under the inflammatory stimulation condition, it can facilitate the production of more nitric oxide (NO) by promoting the inducible nitric oxide synthase (iNOS) expression in microglia (Färber and Kettenmann, 2006; Maksoud et al., 2021). Therefore, we examined the effect of the 1,064-nm laser on the intracellular Ca^2+^ level in microglia to understand whether the light-induced change in the intracellular Ca^2+^ level can impact the polarization of microglial phenotypes or not. Akin to the immunofluorescence experiment, we used the same laser and followed similar fAβ treatments to analyze the different manifestations of intracellular Ca^2+^ level in each group of microglia. The representative confocal microscope images are shown in Figure 4A, and the quantification of the fluorescence measurements is shown in Figure 4B. The fluorescence intensity of Ca^2+^ in the fAβ+1,064-nm laser group was 36.40% lower than that in the fAβ-treated group, whereas the intracellular Ca^2+^ level in the Aβ+1,064-nm laser group was found to be similar to that in the control group, indicating that fAβ can stimulate the change in Ca^2+^ level in microglia. However, there was no significant difference between the 1,064-nm laser group and the control group (4.71%, *p* > 0.5), suggesting that resting microglia and M2 microglia showed a similar Ca^2+^ level.

### 808- and 1,064-nm laser synergistically inhibited fAβ-mediated neurotoxicity in transwell system of microglia and neurons co-cultured

**Figure 5:**
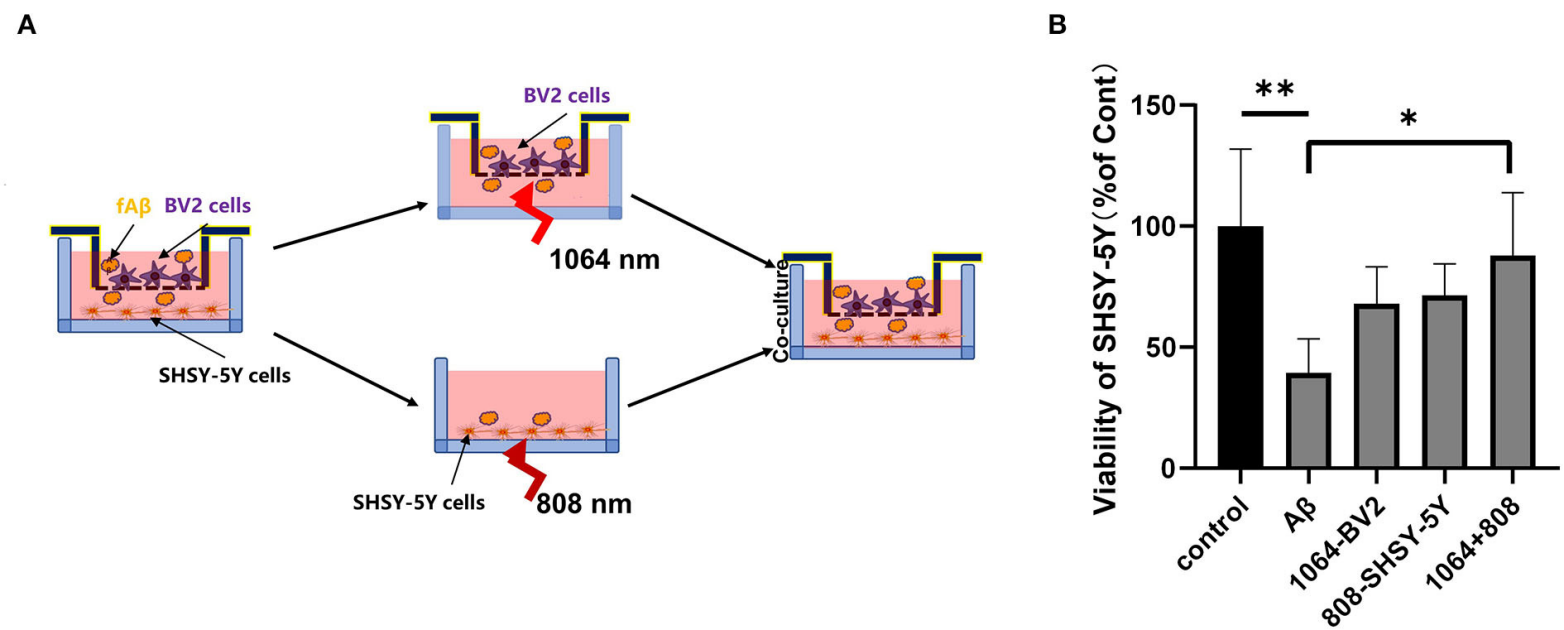
Eight hundred and eight and one thousand and sixty-four-nanometers lasers co-inhibited fAβ-induced neurotoxicity in transwell of AD co-culture model. **(A)** Schematic diagram of PBM irradiation process in the transwell model. **(B)** The viability of SHSY-5Y was tested in five groups with CCK-8 assay: (1) control group, (2) fAβ-treated group, (3) fAβ+1,064-nm laser-irradiated BV2 group, (4) fAβ+808-nm laser-irradiated SH-SY5Y group, and (5) fAβ+808- and 1,064-nm irradiation group. Data in **(B)** are mean ± SEM of at least three independent experiments, *n* = 8, *p < 0.05. The ** symbol indicates the values of *p* < 0.001.

To test whether the 808-nm and 1,064-nm laser irradiations on neurons and microglia have a synergistic effect on the attenuation of Aβ-induced toxicity or not, we tested the viability of SH-SY5Y cells in five different groups under various cell treatments in the co-cultured BV2 and SH-SY5Y cells, placed in the transwell insert system. The irradiation process is shown in Figure 5A. Microglial cells were cultured in the upper chambers, in which the diameter of the membrane hole at the bottom of the chamber was 3μm. BV2 cells were transferred to another aseptic 24-well plate when they were irradiated with the 1,064-nm laser. Then, SHSY-5Y cells were irradiated with 808 nm, whereas BV2 cells were irradiated with the 1,064-nm laser. As shown in Figure 5B, the group of dual-laser (808 and 1,064 nm) irradiations revealed the most significant recovery effect on neuronal viability, which is comparable with the control group (*p* < 0.05), whereas the individual laser treatment groups (*p* > 0.05) exhibited much less neuronal viability. Apparently, the individual recovery of neuronal viability effect was slightly better in the 808-nm laser-irradiated SH-SY5Y cell group than the 1,064-nm laser-irradiated BV2 cell group. It is worth mentioning that the viability of neurons in the fAβ treatment group was the worst, which reconfirmed the fAβ-induced neurotoxicity in SH-SY5Y cells. Although BV2 cells did not have the direct contact with SH-SY5Y cells, it still exerted a protective effect on neurons after returning to the same culture environment (post-PBM treatment), indicating that BV2 cells and SH-SY5Y cells were engaged in the exchange of beneficial substances which in turn facilitated the neutralization of the fAβ-mediated neurotoxicity in the transwell co-culture system.

## Discussion

In this study, the highlight of the finding is that PBM can synergistically decrease the burden of fAβ in the microglia and neuroblastoma culture models by employing the 808- and 1,064-nm lasers, respectively. Basically, it was revealed that the 1,064-nm laser can indirectly eliminate the fAβ-induced neurotoxicity by regulating microglia toward the M2 anti-inflammatory phenotype, leading to the improved fAβ phagocytosis and the elevated release of TGF-β anti-inflammatory factors. Meanwhile, the intracellular Ca^2+^ level is also reliant on the nature of microglial polarization (with M1 or M2 phenotype). Basically, the intracellular Ca^2+^ level in M1 microglia was found to be significantly higher than that in M2 microglia, which implied that the change in the intracellular Ca^2+^ level is directly correlated with the type of microglial polarization and it may transform the microglial function. On the contrary, the neuroblastoma cells were directly regulated to restore their cellular viability using the 808-nm laser. Overall, the coordinated PBM effect of the two types of brain nerve cells in their co-culture system synergistically attenuated the fAβ-induced neuronal toxicity. Our findings demonstrated that the 1,064- and 808-nm lasers can mitigate the fAβ-induced neurotoxicity not only by modulating microglial phenotype but also through the direct enhancement of neuronal activity in the microglia and neuroblastoma co-culture AD models (transwell insert system, approach to AD *in vitro* model). Therefore, the co-regulation of dual-wavelength lasers at 1,064 nm and 808 nm may actually be practically implemented as a novel promising therapeutic strategy for AD.

It was already confirmed that the microglial phenotype can be regulated by PBM. PBM with specified NIR laser may lead to the increased M2 microglial phenotype with a concurrent decrease in the M1 phenotype of microglia. It has been reported that when PBM was carried out with dose-dependent 808-nm laser, it resulted in the polarization of M1 phenotype on high-dose irradiation, but the polarization of M2 phenotype on low-dose irradiation in microglia (von Leden et al., 2013). Similarly, our results also demonstrated that the 1,064-nm laser can regulate the polarization of microglia, preferentially toward the M2 phenotype. In our experiment, CD206 and Arg1 acted as the markers of M2 phenotype microglia and CD68 and iNOS are commonly used as the markers for the inflammatory phenotype of microglia M1 (Xu N. et al., 2017). The immunofluorescence study also validated that in comparison with the control group, the 1,064-nm laser irradiation can far more effectively inhibit the expression of CD68 and iNOS, whereas the same laser leads to the increase in the CD206 and Arg1 expression in microglia. Microglial activation with different phenotypes may produce different cytokines. Studies by Amadio et al. also revealed that the IL-6 expression can be inhibited by the 808-nm laser, when implemented on the brain of aged mice. It was established that the body function of aged mice could be effectively improved by combining the aerobic exercise with the regulation of PBM (Amadio et al., 2015). The upregulated expression of TGF-β was also found to be pertinent in PBM treatment (Vogel et al., 2021). Similarly, our study also validated that the 1,064-nm laser can result in the inhibition of IL-6 expression while persuading the increase in TGF-β production in microglia.

Our study also revealed that the changes in microglial function have a close relation with the changes in the intracellular Ca^2+^ levels. Many studies have already demonstrated that the intracellular Ca^2+^ level can impact the executive functions of microglia such as releasing the inflammatory and anti-inflammatory cytokines, carbon monoxide, and enabling the phagocytosis (Inoue, 2002; McLarnon, 2005; Hanisch and Kettenmann, 2007). However, the majority (80%) of microglial cells showed no spontaneous Ca^2+^ transients at rest and in conditions of strong neuronal activity (Eichhoff et al., 2011). Hoffmann et al. (2003) found that the lipopolysaccharide (LPS)-stimulated microglia can lead to the continuous increase in the intracellular Ca^2+^ level. However, introducing a Ca^2+^ chelator in the system resulted in the significant reduction in the LPS-induced ROS level as well as the amount of pro-inflammatory cytokines, released from microglia. This also suggested that the entry of Ca^2+^ in cells can play an important role in enabling the microglial polarization. It is important to note that some pro-inflammatory factors such as tumor necrosis factor α (TNF-α) and IL-1β can also lead to the increase in the intracellular Ca^2+^ level (Goghari et al., 2000; McLarnon et al., 2001; Franciosi et al., 2002). Activated microglia will be secreted by the above pro-inflammatory factors, which might lead to the further microglial polarization owing to the secretion of negative feedback mechanism. Both the microglial activation and the change in intracellular Ca^2+^ level can impact the transformation of phenotypes toward maintaining central nervous homeostasis. Therefore, exploring the exact relation between the change in the intracellular Ca^2+^ level and the transformation of microglial function could be advantageous to realize the precise mechanism of PBM. The present investigation suggested that fAβ can induce the increase in the intracellular Ca^2+^ level in microglia, which is consistent with the previously reported LPS-induced results (Hoffmann et al., 2003). However, PBM treatment with the 1,064-nm laser irradiation resulted in the reduction in the intracellular Ca^2+^ level in microglia, signifying that the 1,064-nm laser can modulate the microglial phenotype by regulating the intracellular Ca^2+^ level which was corroborated by the immunofluorescence study. Therefore, the fAβ-activated inflammatory M1 microglial cells were effectively polarized to the anti-inflammatory M2 microglia, using this synergistic PBM strategy.

Photobiomodulation can also facilitate the upregulated brain-derived nutritional factors (BDNF) due to its anti-inflammatory and oxidative stress elimination effects (Huang et al., 2013; Duggett and Chazot, 2014). Several studies followed different mechanisms, such as the activation of Akt/GSK3β/β-catenin pathway and PKC pathway, to explore neuronal regulatory effects of PBM (Duan et al., 2003; Zhang et al., 2008, 2012; Liang et al., 2012; Huang et al., 2013, 2014; Duggett and Chazot, 2014). Duggett and Chazot (2014) demonstrated the inhibition of Aβ-induced neuronal apoptosis using the 808-nm laser in CAD glioma cells and Aβ co-culture model. Similarly, in this study, the use of the 808-nm laser at a dose of 10 J/cm^2^ showed the excellent results in protecting the neuroblastoma cells and restoring the neuronal viability. It might be mentioned that few additional mechanisms for PBM of neuronal cells have also been reported in the past. For instance, Meng et al. (2013) achieved the improvement in the neuronal ailment models concerning Aβ-induced dendritic atrophy and tangles in neurons using PBM wherein they elucidated the upregulated BDNF expression in neurons through the ERK/CBEB activation pathway. Some other studies have also attributed the therapeutic outcome of PBM to the reduction in the ROS levels in nerve cells, leading to the subsequent waning of oxidative stress in neurons (Huang et al., 2014; Rupel et al., 2018; Zupin et al., 2019).

The transwell insert system is widely used in studying the crosstalk activities of two different kinds of cells, wherein the influence of one cell line on another kind of cell can be avoided during the cell treatment process. For example, the transwell system, expressing the LPS-treated microglia, was utilized by Fenner et al. (2021) to demonstrate the inflammatory effects of healthy neurons. Song et al. (2012) also cultured microglia and neuroblastoma in the transwell system. Microglia, treated with LPS/632.8-nm laser, were placed in the transwell system and co-cultured with neuron cells. It revealed that PBM treatment can lead to the attenuation of the expression of LPS-induced cytotoxicity. In this study, also the transwell co-culture AD system was established by treating microglial and neuroblastoma cells with fAβ, wherein the two different kinds of cells (microglia and neuroblastoma) were exposed to the 1,064- and 808-nm lasers, respectively. Moreover, the two kinds of cells were cultured and grown in the same environment without blocking the crosstalk between them. Cytokines such as IL-6 and TGF-β, secreted by microglia, were cultured in the upper layer of the transwell system which directly stimulated the underlying neuroblastoma cells, and the synergistic PBM effect of the 808-nm and 1,064-nm laser irradiations showed the best therapeutic results (Figure 5), even reaching the level of the control group.

The choices of wavelength are vital to PBM. The most mentioned mechanisms of PBM are the absorption of photons by CCO. The higher the activity of CCO, the more oxygen and metabolic energy the mitochondria consume. The structure of CCO contains two heme iron and three copper centers with different absorption spectra. Different wavelengths of photons have different effects on CCO, and 810-nm (similar to 808 nm) lights have been shown to increase CCO activity (Sanderson et al., 2018). Because neurons are highly dependent on oxygen metabolism, this photobiological stimulation mechanism leads to changes in neuron metabolism, such as an increase in ATP and the modulation of nitric oxide and Ca^2+^ levels, facilitating the viability of neurons (Gonzalez-Lima and Auchter, 2015). Huang et al. reported that the 810-nm lasers could promote ATP production, regulate mitochondrial membrane potential and NO releases of neurons, and result in improving neuronal viability (Huang et al., 2014). Another study also showed that ATP levels in the 808-nm laser-treated neurons were significantly higher than those in the untreated groups (Oron et al., 2007). The better mitochondrial activity provides a positive feedback on neuron function and vitality. In addition, the effects of the 808-nm lasers on lipid metabolism of neurons were investigated by a CARS microscope, and it was found that the lipid and ROS level of neurons increased irradiated by the 808-nm lasers (Levchenko et al., 2019). In the state of cellular stress, the increased lipid level can play a protective role on cells (Lee et al., 2013). This may be one of the underlying mechanisms by which neurons can be activated by the 808-nm lasers after toxic stimulation of Aβ. In conclusion, the 808-nm lasers can stimulate the increase in ATP level in neurons and play a protective role in neurons under oxidative stress. On the contrary, some studies have employed lights with wavelengths similar to 1,064 nm in animal AD models to improve memory and reduce the burden of Aβ plaques on the cerebral cortex of AD mice (Michalikova et al., 2008; Grillo et al., 2013). However, the exact underlying mechanism of 1,064-nm lights on AD remains unclear. In a recent study, it has also been demonstrated in an AD mouse model to reduce the M1 phenotype microglia, thus promoting the phagocytosis ability of microglia to Aβ plaques (Tao et al., 2021). Our study also proved that the 1,064-nm lasers could regulate the phenotype of microglia, reduce the secretion of inflammatory cytokines and promote the secretion of anti-inflammatory cytokines, and change the levels of intracellular Ca^2+^ of microglia.

**Figure 6:**
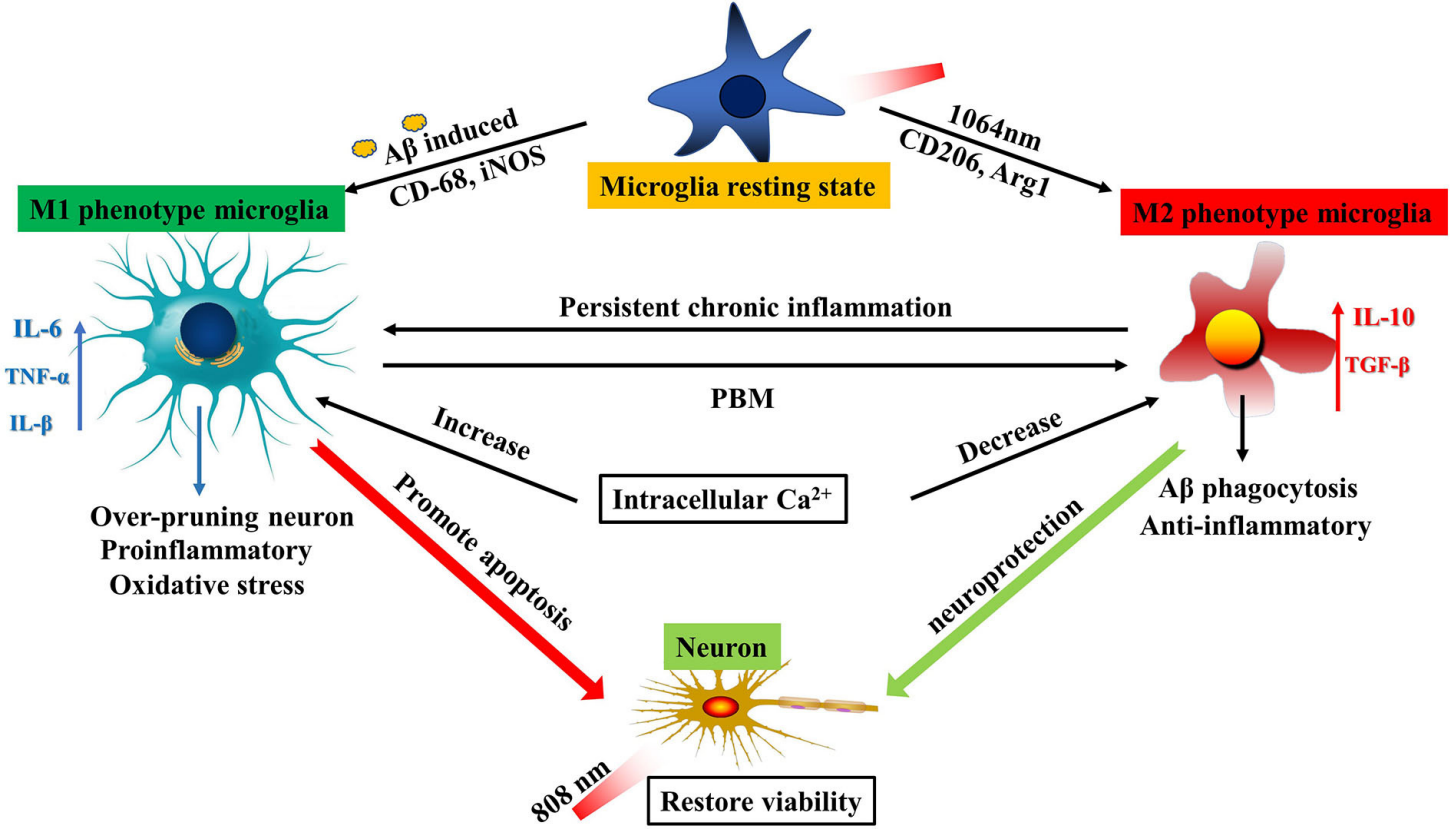
Schematic diagram of PBM dual-wavelength synergistic regulation of microglia and neurons. On the one hand, microglia can be activated to be M1-like phenotype by fAβ and to be M2-like by the 1064-nm laser. An increase in the intracellular Ca^2+^ level led to polarization of M1 microglia, and inflammatory factors such as IL-6 and TNF-α were also upregulated, resulting in promoting neuronal apoptosis. However, this mechanism has the opposite performance under the regulation of PBM. The reduced intracellular Ca^2+^ level led to the upregulation of anti-inflammatory factors such as IL-10 and TGF-β in M2 cells. The result is neuroprotection. On the other hand, the 808-nm laser directly protects the neuronal activity. The synergistic working mode can maximize the therapeutic effect of PBM by acting on the two kinds of cells with important functions in the AD model.

In conclusion, our results demonstrated that the 1,064- and 808-nm lasers can regulate microglia and neurons, respectively, to resist the fAβ-induced neurotoxicity. A reduction in the intracellular Ca^2+^ level in microglia using the 1,064-nm laser resulted in not only the anti-inflammatory expression with TGF-β factor, but also regulating the polarization of microglia toward the M2 phenotype. On the contrary, PBM with the 808-nm laser directly caused the enhancement of neuronal viability (Figure 6). Overall attenuation of the fAβ-induced neurotoxicity was maximum when the two PBM approaches (with two different lasers 1,064 and 808 nm) were combined to regulate the transwell co-culture models for both cell types. It also provided a valuable reference to understand the mechanism of the microglial activation in the AD model upon the 1,064-nm laser irradiation, which is advantageous for the further exploration of PBM in AD treatment with diverse optimal wavelengths and doses of the light parameters.

## Materials and methods

### Experiment groups

The immunofluorescence, ELISA assay, and cellular Ca2+ determination experiments were divided into four groups: (a) control group, (b) fAβ treatment group, (c) 1,064-nm laser-irradiated group, and (d) the fAβ+1,064-nm laser-irradiated group. After fAβ was added to the cell lines and cultured for 24 h, the cells were irradiated with 1,064-nm (10 J/cm^2^) laser light.

The transwell system experiment was divided into five groups: (a) control group; (b) fAβ treatment group; (c) fAβ+SHSY-5Y cells irradiated with the 808-nm laser group; (d) fAβ+BV2 cells irradiated with the 1,064-nm laser group; and (e) fAβ+ (BV2 cells irradiated with the 1,064-nm laser and SHSY-5Y cells irradiated with the 808-nm laser group). The cell viability of each experimental group was tested with CCK-8 reagent. Microglia and neurons in all groups were cultured together for 24 h after PBM, and then, the viability of neuroblastoma was measured.

### PBM therapy

**Figure 7:**
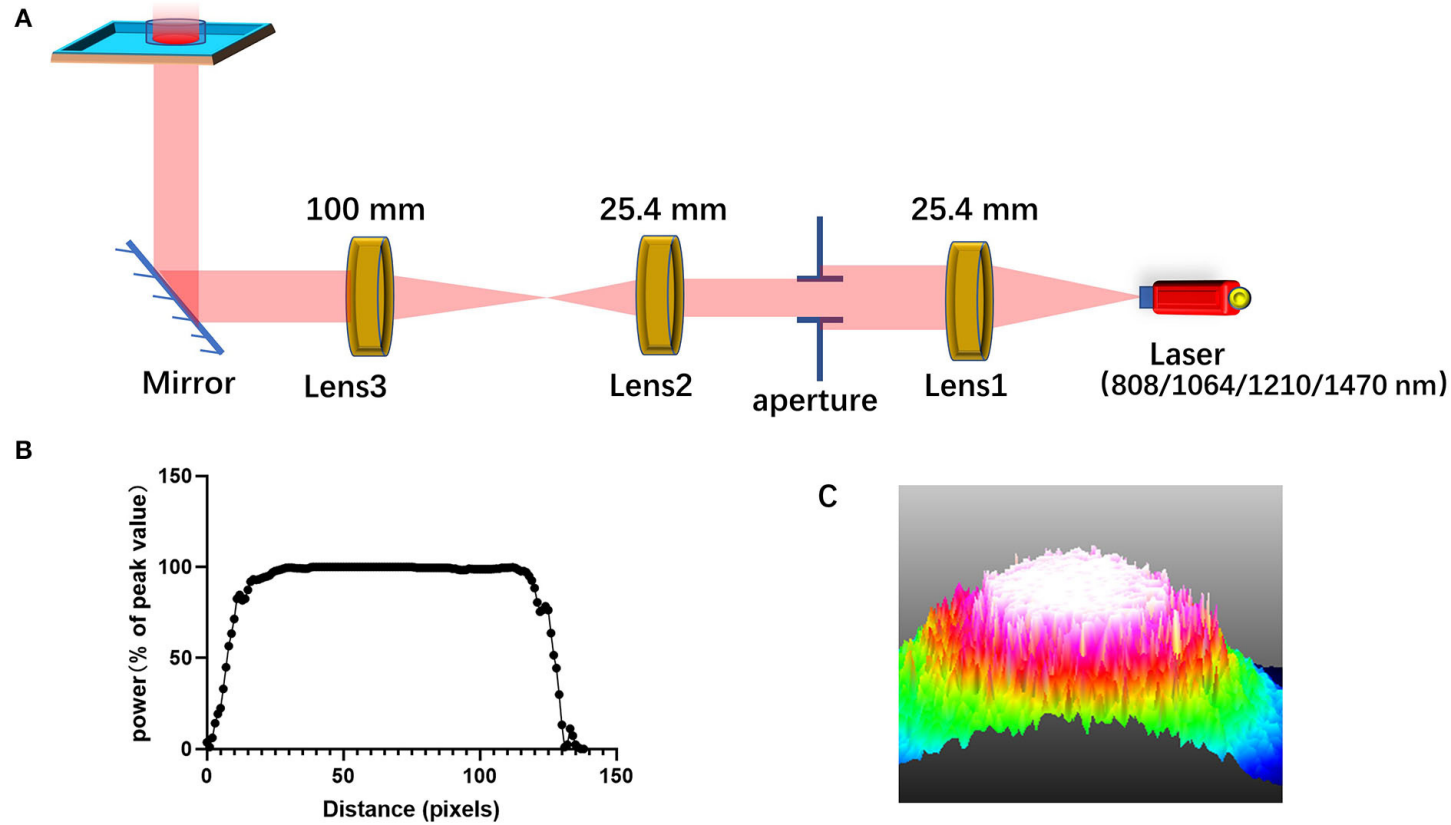
Schematic diagram for laser irradiation apparatus. **(A)** A diagram of the system that homogenizes the intensity distribution of light beam; **(B)** the intensity distribution of the homogenized spot; **(C)** 3D light intensity distribution as shown on the beam quality analyzer.

The schematic representation of the laser irradiation is shown in Figure 7A. To ensure that the power density irradiated to the cells is uniform and avoid the power attenuation when the laser propagates in the medium, the light output from laser combiner was expanded and collimated before irradiating the cells from the bottom of the cell culture dish or aperture plate. The aperture can adjust the size of the light spot. Figure 7C shows the light intensity distribution of the light spot obtained by using the beam quality analyzer (Thorlabs). Through this lighting system, the power distribution of the light spot on the sample has been made uniform, as shown in Figure 7B. Light absorption by the medium is avoided by irradiating the petri dish from the bottom.

### Cells culture

BV2 cells (mouse microglia) were cultured at 37°C, under a 5% CO_2_ atmosphere in DMEM medium with 10% fetal bovine serum and 1% penicillin–streptomycin solution. SHSY-5Y cells (immortalized human neuroblastoma) were also cultured at 37°C, under a 5% CO_2_ atmosphere in DMEM/F12 medium with 15% fetal bovine serum and 1% penicillin–streptomycin solution. The cells were passaged when growing up to 80% confluence. To activate microglia, we stimulated cells with fAβ (1 μ*M*) before PBM. BV2 and SHSY-5Y cells were planted in the upper and lower chambers of the transwell of 12 wells (Corning, 3462), respectively, which were then cultured in the same medium, used for SH-SY5Y cells.

### Aβ incubation

Beta-amyloid (1–42) and HiLyte Fluor 555-labeled and non-labeled human Aβ_1−42_ (1 mg) peptide were purchased from AnaSpec and Aladdin, UK, respectively. Prior to use, 1 mg freeze-dried powder of Aβ_1−42_ and hexafluoroisopropanol (HFIP) were placed on ice for pre-cooling. Two hundred and twenty-two microliters of HFIP was injected into the reagent bottle, sealed and mixed gently, and kept at room temperature for 60 min until the liquid became clear to obtain the Aβ-HFIP solution (1 mM). Four sterile 1.5-ml EP tubes were taken, and the Aβ-HFIP solutions, divided into four equal parts (55 μL each), were individually placed in each tube. HFIP was dried by a vacuum freeze-drying apparatus, and an Aβ peptide film was obtained, which was then stored at −20°C. In a separate tube, 11 μL DMSO was added to the Aβ peptide membrane. After 10 min of water bath ultrasound (power 300 W, frequency 35 Hz), Aβ-DMSO solution (5 mM) was obtained. The pre-cooled 539 μL PBS solution (100 μM) was added to Aβ-DMSO solution and mixed gently. To further promote the formation of the fibrils, the solution was incubated at 37°C for 1 week. Prior to use, it was diluted 100 times to 1μM in the medium and then cultured with cells.

### Immunofluorescence

The cells growing on slides were fixed with 4% of paraformaldehyde, washed three times with PBS, and then permeated with 0.5% Triton X-100 (diluted with PBS) for 20 min at room temperature. After washing the slides three times with PBS, 1% bovine serum albumin (BSA) as a blocking agent was cultured with cells at room temperature for 30 min. Then, absorbing the sealed solution with absorbent paper, a sufficient amount of anti-CD68 (M1 marker, ab237968, Abcam), anti-iNOS (M1 marker, ab49999, Abcam), anti-CD206 (M2 marker, ab64693, Abcam), anti-arginase1 (M2 marker, ab91279, Abcam), and anti-insulin degrading enzyme (ab32216, Abcam) primary antibody, diluted with BSA (diluted 1,000 times to 1 μM), was added to each slide drop and placed in a wet box, which was incubated at 4°C overnight. Next, the goat anti-rat IgG H&L Alexa Fluor 488-conjugated (ab150157, Abcam), donkey anti-rabbit IgG H&L-conjugated (Alexa Fluor® 488) (ab150073, Abcam), goat anti-mouse IgG H&L-conjugated (Alexa Fluor 594) (ab150116, Abcam), and donkey anti-rabbit IgG H&L Alexa Fluor 647-conjugated (ab150075, Abcam) secondary antibodies were used for labeling. The cells were imaged with a laser scanning confocal microscope. The average fluorescence intensity of 20 representative cells was obtained for quantitative analysis.

### Determination of intracellular Ca^2+^ level

After 24 h of incubation with fAβ in the fAβ group and the fAβ+1,064-nm laser group, the latter group was irradiated with the 1,064-nm laser. Then, it was returned to the incubator culture for 1 h. The Fura2-Am Ca^2+^ probe was diluted 1,000 times in the medium to obtain 2 μM solution. Prior to imaging, this 2 μM Fura2-Am Ca^2+^ probe solution was added to a cell petri dish and cultured for 10 min followed by washing three times with PBS. The cells were imaged with a laser scanning confocal microscope. The average fluorescence intensity of 20 representative cells was obtained for quantitative analysis.

### ELISA assay

Prior to testing, the culture medium of BV2 cells was collected. Then, IL-6 and TGF-β1 (ab222503 and ab119557, Abcam) ELISA kits were employed for assessing the cytokine expression profiles of activated or non-activated microglia. The cytokine profiles of microglia were determined after 3-h PBM irradiation. The supernatants of culture medium of microglia were processed by the ELISA method according to the manufacturer's protocol. The absorbance of the resulting solution at 450 nm for each well was measured by a microplate reader. The concentrations of the cytokines in the samples were analyzed on Excel software.

### Flow cytometry

To quantitatively determine the amount of microglial phagocytosis of fAβ plaques, microglia were collected and used for flow cytometry measurement, wherein to measure the fluorescence in each sample with at least 10,000 cells, the amount of fluorescent fAβ was analyzed.

### Cell viability measurement

Cell viability was measured by CCK-8 assay according to the manufacturer's protocol. SHSY-5Y cells were plated on 24-transwell plates or the 24-well plates (500 μ*l*, 1 × 10^5^/well) after PBM. The 50-μL CCK-8 solution was added to each well, and the cells were incubated for 2 h in the incubator. The absorbance of the resulting solution at 450 nm for each well was measured by a microplate reader (Synergy H1).

### Statistical analysis

Comparisons for normally distributed data with three or more groups were tested by one-way ANOVA Dunnett's test. Comparisons for normally distributed data with two groups were analyzed by two-tailed unpaired *t*-tests. The difference between the two means was considered to be statistically significant when *P* was < 0.05. The results are expressed as the mean ± SEM.

## Data availability statement

The original contributions presented in the study are included in the article/supplementary material, further inquiries can be directed to the corresponding author/s.

## Author contributions

RZ, TZ, and JQ conceptualized the study. RZ and TZ performed the analysis of data. RZ performed the data collection and wrote the original draft. SS, ZL, SL, HX, and JQ reviewed and edited the manuscript. JQ reviewed and supervised the writing of the manuscript. All authors contributed to the article and approved the submitted version.

## Funding

This work was partially supported by the National Natural Science Foundation of China (61835009/62127819) and Shenzhen International Cooperation Project (GJHZ20190822095420249).

## Conflict of interest

The authors declare that the research was conducted in the absence of any commercial or financial relationships that could be construed as a potential conflict of interest.

## Publisher's note

All claims expressed in this article are solely those of the authors and do not necessarily represent those of their affiliated organizations, or those of the publisher, the editors and the reviewers. Any product that may be evaluated in this article, or claim that may be made by its manufacturer, is not guaranteed or endorsed by the publisher.
