# Supplementary material for: Synergistic photobiomodulation with 808-nm and 1064-nm lasers to reduce the β-amyloid neurotoxicity in the in vitro Alzheimer's disease models
Source: Front Neuroimaging. 2022 Jul 22;1:903531. doi: 10.3389/fnimg.2022.903531 (PMC10406259; doi:10.3389/fnimg.2022.903531)
Supplement: Supplementary file 1 [file Table_1.DOCX]

### Section1


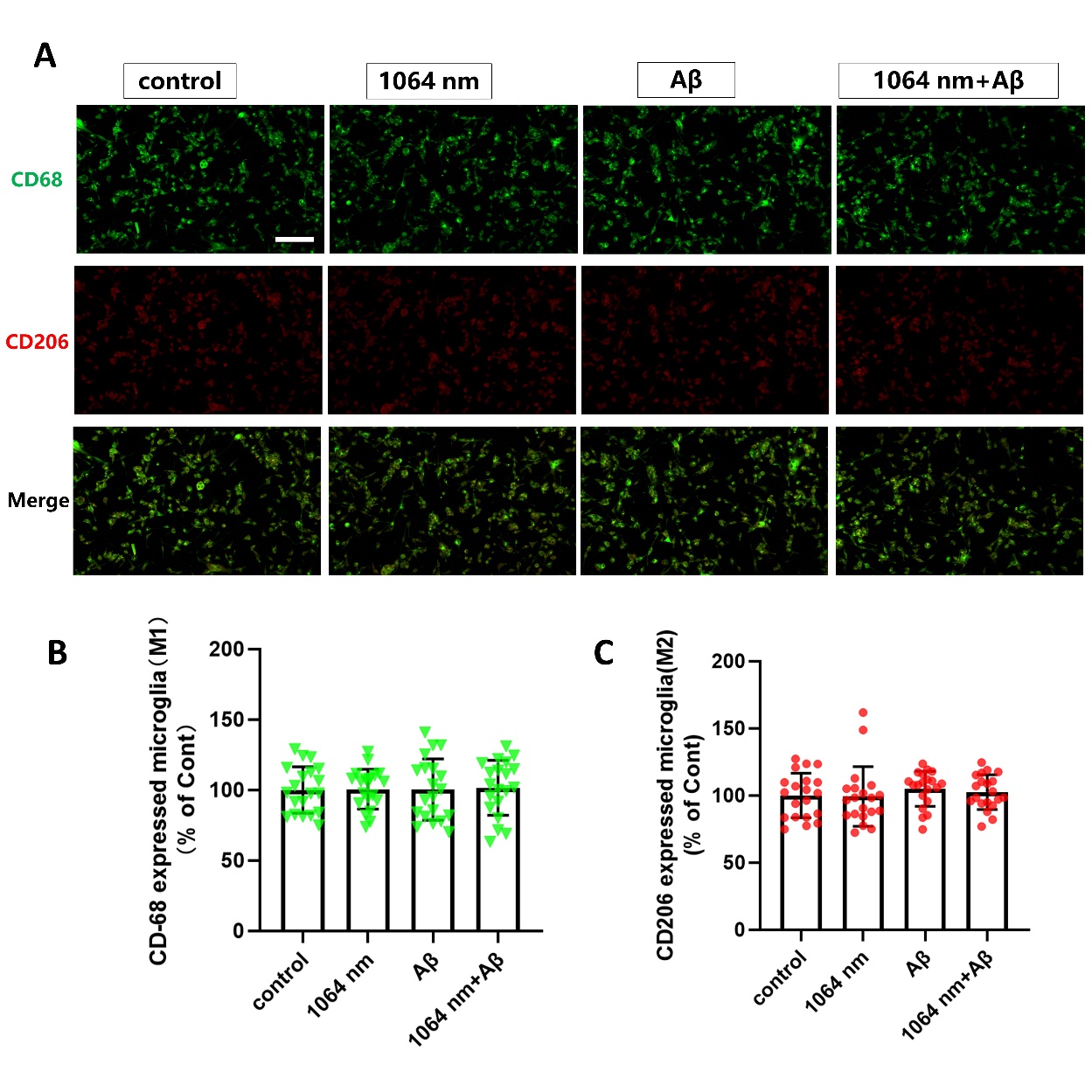


**Figure. The PBM regulations of microglia phenotype were inhibited by BAPTA-am and Ca^2+^ free buffer pretreatments.** (A) The fluorescence imaging of CD206 and CD68 after BAPTA-AM and Ca^2+^ free buffer pretreatments, scale bar,100$\mu m$. (B, C) quantification of the fluorescence measurements. All data are normalized to the control group and shown as the mean ± SEM of at three independent experiments.

To determine whether intracellular Ca^2+^ plays a role in phenotype regulation of microglia, we performed studies to examine the effects of an in tricellular Ca^2+^ chelator (BAPTA-AM solution) and a Ca^2+^ free environments, on expression of CD206 and CD68 in microglia. Prior to PBM or fAβ treatments, microglia cells incubated with 5$\mu M$ BAPTA-AM and Ca^2+^ free buffer for 20 minutes at 37$℃$ and washed twice with PBS solution. As shown in the figure above, there were no significant differences in the expression of CD206(M2 microglia marker) and CD68 (M1 microglia marker) between the three experimental groups and the control group in microglia. Therefore, the intracellular Ca^2+^ level plays a key role in the polarization of microglia in the PBM process.
